# The Impact of Bacillus Calmette-Guérin (BCG) Revaccination on COVID-19 Infection Among Healthcare Workers: A Systematic Review and Meta-Analysis

**DOI:** 10.7759/cureus.99986

**Published:** 2025-12-24

**Authors:** Hosam Hadi Hassan Awaji, Rayan N Sahli, Fawzyh B Albalwi, Wasayef S Albalawi, Teef A Muhawish, Hana M Albalawi, Amal K Alsubiti, Jawaher S Alanazi, Abeer Hamdi, Nabiah Alshehri, Fawziyah S Qarni, Nouf Abu Salem, Fida N Albalawi

**Affiliations:** 1 Preventive Medicine, North West Armed Forces Hospitals, Tabuk, SAU; 2 Preventive Medicine, King Salman Armed Forces Hospital, Tabuk, SAU; 3 Ophthalmology Nursing, King Salman Armed Forces Hospital, Tabuk, SAU; 4 Nursing, King Salman Armed Forces Hospital, Tabuk, SAU; 5 Medicine, University of Tabuk, Tabuk, SAU; 6 Radiosurgery, King Salman Armed Forces Hospital, Tabuk, SAU; 7 Medical Education and Simulation, King Salman Armed Forces Hospital, Tabuk, SAU; 8 Preventative Medicine/Nursing, King Salman Armed Forces Hospital, Tabuk, SAU; 9 Ophthalmology, King Salman Armed Forces Hospital, Tabuk, SAU

**Keywords:** bcg, covid-19 prevention, healthcare workers, revaccination, vaccine

## Abstract

The Bacillus Calmette-Guérin (BCG) vaccine has been hypothesized to confer nonspecific immune protection against viral infections, including coronavirus disease 2019 (COVID-19). This meta-analysis evaluates the protective role of BCG vaccination in preventing COVID-19 among healthcare workers (HCWs). A systematic review and meta-analysis were conducted of randomized controlled trials (RCTs) that reported the effect of BCG vaccination for COVID-19 prevention in HCWs compared with placebo. Nine RCTs were included, with a total of 10,295 HCWs. Pooled odds ratios (ORs) and mean differences (MDs) were calculated using random- and fixed-effects models to assess the impact on symptomatic COVID-19, severe disease, hospitalization, seropositivity, duration of illness, and serious adverse events. Heterogeneity was evaluated using I² statistics.

BCG vaccination did not significantly reduce the risk of symptomatic COVID-19 (OR = 1.05, 95% CI: 0.94-1.17, p = 0.43, I² = 47%), severe COVID-19 (OR = 1.20, 95% CI: 0.97-1.48, p = 0.09, I² = 0%), or hospitalization (OR = 1.04, 95% CI: 0.71-1.50, p = 0.86, I² = 0%). There was no significant difference in the duration of symptomatic illness (MD = 0.09 days, 95% CI: -1.41-1.59, p = 0.90, I² = 80%) or in rates of COVID-19 seropositivity (OR = 1.27, 95% CI: 0.81-1.98, p = 0.30, I² = 76%). The incidence of serious adverse events was not significantly different between groups (OR = 1.43, 95% CI: 0.34-5.97, p = 0.62, I² = 81%). This meta-analysis found no significant protective effect of BCG vaccination against any clinical or serological endpoint of COVID-19 in HCWs. The results do not support the use of BCG as a preventive measure against COVID-19 in this population.

## Introduction and background

The Bacillus Calmette-Guérin (BCG) vaccine, originally developed for tuberculosis (TB) prevention, has long been recognized for its nonspecific immune-enhancing effects that protect against various respiratory infections. This phenomenon, often referred to as trained immunity, is mediated by epigenetic and metabolic reprogramming of innate immune cells, leading to enhanced immune responses against unrelated pathogens. In recent years, there has been growing interest in the potential role of BCG vaccination in mitigating the impact of viral infections, including coronavirus disease 2019 (COVID-19) [1].

The COVID-19 pandemic, caused by the severe acute respiratory syndrome coronavirus 2 (SARS‑CoV‑2) virus, has placed an enormous burden on global healthcare systems, with healthcare workers (HCWs) being particularly vulnerable due to their continuous exposure to infected patients. As a result, strategies to reduce the severity and incidence of COVID-19 in HCWs have been a priority [2]. Early epidemiological observations suggested a correlation between countries with routine BCG vaccination programs and lower COVID-19 incidence and mortality rates, leading to the hypothesis that BCG vaccination may confer some level of protection against SARS-CoV-2. Consequently, several clinical trials were initiated to evaluate the efficacy of BCG revaccination in preventing COVID-19, particularly among high-risk populations such as HCWs [3].

BCG revaccination has been proposed as a potential strategy to boost immune responses in individuals already vaccinated in their infancy. This approach is based on evidence that revaccination enhances trained immunity, leading to increased resistance against infections beyond TB [4]. Some observational studies and randomized controlled trials (RCTs) have explored whether BCG revaccination could lower the risk of COVID-19 infection, reduce symptom severity, or improve clinical outcomes in HCWs. However, findings have been inconsistent, with some studies reporting significant protective effects, while others have found no substantial benefit [5].

Given the conflicting results in the literature, systematic review and meta-analysis are warranted to comprehensively assess the impact of BCG revaccination on COVID-19 incidence, severity, and outcomes among HCWs [6]. By synthesizing data from multiple studies, this meta-analysis aims to provide a clearer understanding of whether BCG revaccination can be considered an effective preventive measure against COVID-19 in this high-risk population. The findings of this study may have important implications for global public health policies, particularly in resource-limited settings where access to specific COVID-19 vaccines remains a challenge.

This meta-analysis addressed key research questions, including: 1. Does BCG revaccination reduce the incidence of COVID-19 in HCWs? 2. Does it mitigate disease severity and improve clinical outcomes? 3. What are the immunological mechanisms underpinning the potential protective effects of BCG against COVID-19? By answering these questions, this study aimed to contribute valuable insights into the potential repurposing of BCG revaccination as a supplementary tool in the fight against COVID-19 and future viral pandemics.

## Review

Methods

The conduct and reporting of this meta-analysis were performed in accordance with the principles of the Cochrane Handbook for Systematic Reviews of Interventions, version 6, and the Preferred Reporting Items for Systematic Reviews and Meta-Analyses (PRISMA) guidelines [7].

Research Question

Does BCG revaccination reduce the incidence of COVID-19 in HCWs, mitigate disease severity, and improve clinical outcomes?

Research Aim

This meta-analysis aimed to evaluate the role of BCG revaccination in reducing the incidence and severity of COVID-19 among HCWs.

Research Objectives

To systematically assess the impact of BCG revaccination on the incidence of COVID-19 among HCWs. To evaluate whether BCG revaccination reduces the severity of COVID-19 symptoms and improves clinical outcomes in infected HCWs. To identify potential public health implications of BCG revaccination as a supplementary strategy in pandemic preparedness and response.

Inclusion Criteria

Types of studies: This systematic review and meta-analysis included RCTs that assess the efficacy of BCG revaccination in preventing COVID-19 infection and reducing disease severity in HCWs that were published in English from inception to February 22, 2025.

Participants: The study population included HCWs: physicians, nurses, paramedics, and other frontline medical staff exposed to COVID-19 patients. No restrictions were made with regard to age, sex, or race of the participants.

Interventions: This meta-analysis focused on HCWs who received BCG revaccination. The intervention group (BCG-revaccinated individuals) was compared to control groups that did not receive vaccination and received a placebo.

Exclusion Criteria

Cross-sectional studies, case reports, case series, narrative reviews, editorials, commentaries, or conference abstracts without full-text availability, studies without a control or comparator group (e.g., single-arm interventions), or animal studies or in vitro research were excluded.

Search Strategy

Electronic searches: The following electronic databases were searched for eligible studies: MEDLINE/PubMed, Cochrane Central Register of Controlled Trials (CENTRAL), Web of Science, ProQuest, and Scopus. The search included all articles published in English from inception through February 22, 2025.

The following search terms were used: ((COVID-19) OR (SARS-CoV-2) OR (2019-nCoV) OR (Coronavirus Disease 2019) OR (Post-COVID syndrome) OR (Long COVID)) AND ((BCG vaccine) OR (Bacillus Calmette-Guérin vaccination)) AND (healthcare workers). We used no filters by language or publication period. 

Other resources: The first reviewer searched within the reference lists of obtained articles for other potentially relevant studies that were not retrieved by the electronic search.

Selection of Studies

The first reviewer screened the retrieved reports for eligibility through title and abstract, and full-text screening. The second reviewer checked the retrieved studies, and discrepancies were solved through discussion with a third reviewer.

Data Extraction

The first reviewer carried out data extraction from the included studies using a standardized data sheet which included: (a) the study characteristics (the author, year, the country, design); (b) patient characteristics (age at the time of treatment, sex, sample size, comorbidities); (c) intervention (BCG strain, route of administration, placebo type, follow up duration); (d) the outcomes; number of participants with symptomatic COVID-19, number of participants with severe COVID-19, rate of hospitalizations, number of days with symptoms, serious adverse events, and rate of seropositivity. The second reviewer checked the collected data for consistency and clarity. Any disagreements were settled by referring to the third reviewer.

Measured Outcomes

The measured outcomes were as follows: number of participants with symptomatic COVID-19 (event and total); number of participants with severe COVID-19 (event and total); rate of hospitalization (event and total); number of days with symptoms (mean and standard deviation (SD); serious adverse events (event and total); and number of participants with seropositivity (event and total)

Assessment of Risk of Bias in Included Studies

The risk of bias (ROB) in the included studies was assessed using the National Institute for Health and Care Excellence (NICE) checklists for randomized controlled clinical trials [8].

Data Synthesis

**Figure 1:**
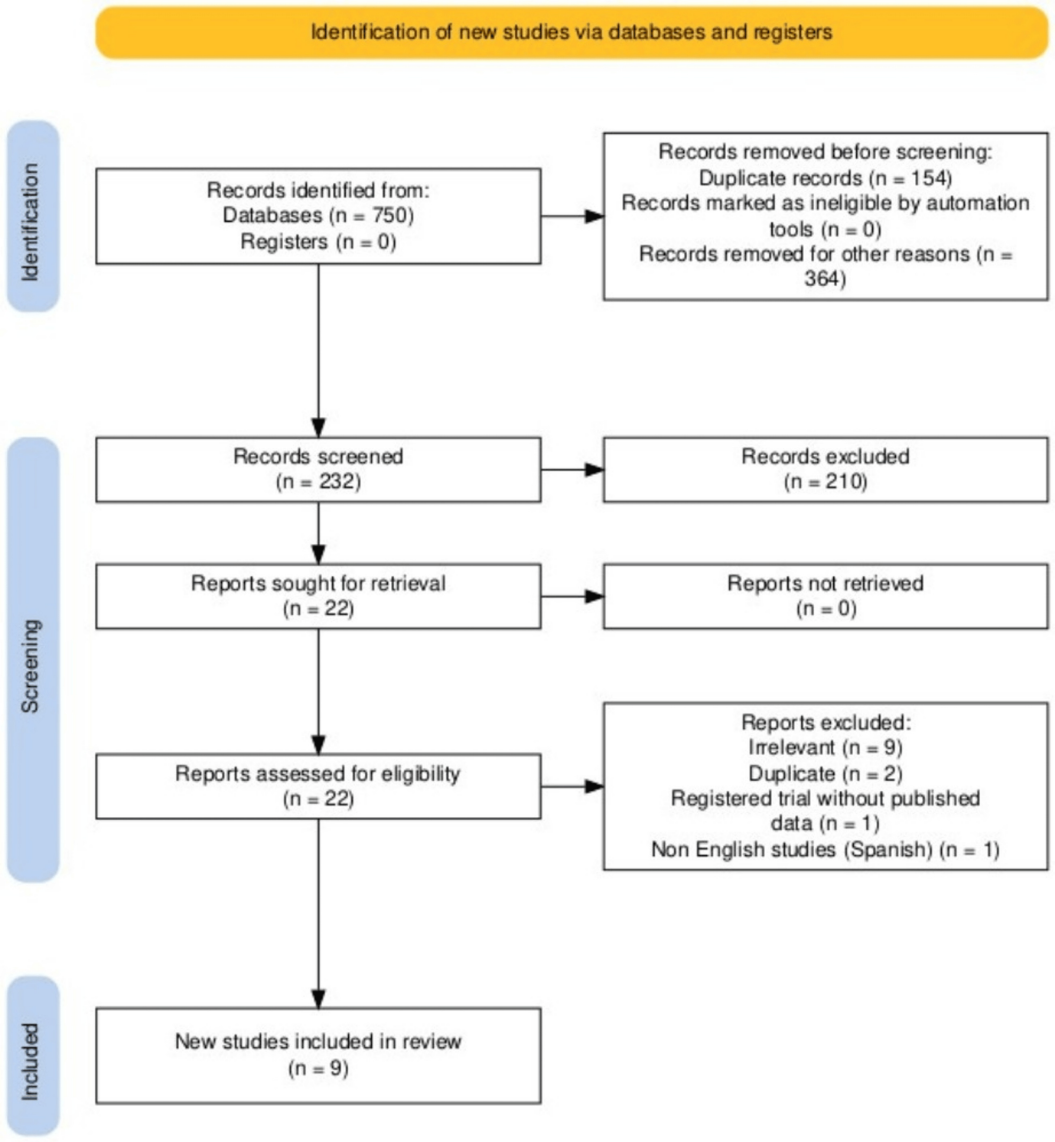
PRISMA flow chart depicting the selection of studies PRISMA: Preferred Reporting Items for Systematic Reviews and Meta-Analyses

Initially, 750 records were retrieved from electronic database searches. After removing duplicates and excluded studies, 22 studies were deemed eligible, of which nine studies (10,307 HCWs) were included in the meta-analysis (Table 1) [9-17], while the other 12 were excluded from the meta-analysis due to irrelevance (n = 9), duplication (n = 2), being in Spanish (n = 1), or being a trial (n = 1). A PRISMA flow chart illustrating the selection of studies is presented in Figure 1 [18].

**Table 1:** Summary of the included studies BCG: Bacillus Calmette-Guérin; SD: standard deviation; RCT: randomized controlled trial; IQR: interquartile range; NM: not mentioned; COVID-19: coronavirus disease 2019; RTIs: respiratory tract infections

Study	Year	Country	Trial design	Sample size (BCG)	Sample size (placebo)	Age (mean ± SD) (BCG)	Age (mean ± SD) (placebo)	Female (%) (BCG)	Female (%) (placebo)	Comorbidities (%) (BCG)	Comorbidities (%) (placebo)	BCG strain	Placebo	Administration	Primary outcomes	Secondary outcomes	Follow-up duration
Anjos Dos et al. [9]	2022	Brazil	Unicentric, parallel, randomized, phase II clinical trial	64	67	41.8 ± 11.0 years	44.2 ± 11.3 years	68.80%	83.60%	17.20%	20.9%​	Mycobacterium BCG Moscow	No placebo was used; the control group was unvaccinated	Intradermal injection in the upper right arm	Reduction of COVID-19 positivity through serological and molecular tests; reduction of COVID-19 symptoms​	Innate immune activation (NK cell activation)​	180 days
Claus et al. [10]	2023	Netherlands	Multicenter, double-blind, randomized, placebo-controlled trial	665	644	41.8 ± 12.7 years	43.2 ± 12.7 years	75.40%	73.40%	Cardiovascular disease: 2.0%, asthma: 7.2%	Cardiovascular disease: 2.6%, asthma: 6.7%	Mycobacterium bovis BCG Danish strain 1331	Normal saline solution	Intradermal injection in the left upper arm	Absenteeism for any reason	Participant-reported positive SARS-CoV-2 tests; symptomatic respiratory infections​	12 months
Czajka et al. [11]	2022	Poland	Multi-center, randomized, double-blind, placebo-controlled phase III clinical trial	168	174	43.8 ± 11.8 years	43.8 ± 11.8 years	77.80%	75.60%	NM	NM	BCG-10 (Biomed Lublin S.A., Poland)	Saline	Single 0.1 mL intradermal injection	Incidence of SARS-CoV-2 infection confirmed by PCR	Number of SARS-CoV-2 infections based on tuberculin test groups; number of infections in different healthcare worker professions	NM
Ten Doesschate et al. [12]	2022	Netherlands	Parallel, double-blind, placebo-controlled randomized trial​	753	758	41.3 ± 12.6 years	42.8 ± 12.7 years	76.00%	72.6%​	Cardiovascular disease: 2.0%, asthma: 7.2%	Cardiovascular disease: 2.5%, asthma: 6.2%	Mycobacterium bovis BCG Danish strain 1331​	Normal saline solution​	Intradermal injection in the left upper arm​	Reduction in healthcare worker absenteeism for any reason	Documented COVID-19 cases, self-reported acute respiratory symptoms, or fever	Median 357 days
Carrero Longlax et al. [13]	2025	United States (Texas and California)	Double-blind, placebo-controlled, RCT	263	266	47.01 ± 12.30 years	46.60 ± 12.40 years	63.50%	68.0%​	21.3% (hypertension), 27.4% (hypercholesterolemia)	19.9% (hypertension), 20.3% (hypercholesterolemia)​	TICE BCG (Merck, USA)	0.9% NaCl saline	Single 0.1 mL intradermal injection	Symptomatic COVID-19 incidence	Asymptomatic SARS-CoV-2 infection, BCG-mediated immune response (cytokine levels), DNA methylation changes	NM
Madsen et al. [14]	2024	Denmark	Single-blinded, placebo-controlled, randomized trial​	610	611	48 (37-56) years	47 (36-57) years	83%	83%	Chronic disease: 36%, cardiovascular disease: 6%, lung disease: 4%, diabetes: 1%	Chronic disease: 32%, cardiovascular disease: 5%, lung disease: 6%, diabetes: 1%	Mycobacterium bovis BCG Danish strain 1331	Normal saline solution	Intradermal injection in the right upper arm​	Reduction in unplanned absenteeism	Verified COVID-19 cases, all-cause hospitalization, and infectious disease episodes​	6 months
Messina et al. [15]	2024	Australia, Netherlands, Spain, United Kingdom, Brazil	Phase III double-blind RCT	1999	1989	Not explicitly stated for each group, but age stratification was done (<40 years, 40-59 years, ≥60 years)		73%	76%	36%	34%	BCG-Denmark (AJ Vaccines, Danish strain 1331)	Saline	Single 0.1 mL intradermal injection in the deltoid​	Symptomatic COVID-19 incidence​; severe COVID-19 incidence	Any COVID-19 (symptomatic or severe), number of COVID-19 episodes, days with symptoms, hospitalizations, oxygen therapy requirement, admission to critical care, asymptomatic SARS-CoV-2 infection​	NM
Dos Santos et al. [16]	2023	Brazil	Phase II-B, multicenter, double-blind, RCT	134	130	Not explicitly stated, but age groups included (18-24, 25-39, 40-59, ≥60)		76.90%	82.30%	21.60%	27.7%​	Moreau (Brazil) or Moscow (India) strains	0.9% NaCl saline	Single 0.1 mL intradermal injection	Cumulative incidence of COVID-19 within 6 months; reduction in signs/symptoms of COVID-19; absenteeism in healthcare workers​	BCG-mediated immune response (antibody levels), adverse events	NM
Upton et al. [17]	2022	South Africa	Phase III, double-blind, randomized, placebo-controlled trial​	500	500	Median (IQR) = 39 (30–49) years	Median (IQR) = 39 (30-50) years	69.80%	71.00%	Hypertension (16.4%), asthma (7.8%), diabetes (6.4%), cardiovascular disease (2.8%), COPD (0.6%)	Hypertension (18.4%), asthma (5.8%), diabetes (6.2%), cardiovascular disease (2.0%), COPD (0.2%)	BCG-Denmark (Statens Serum Institut, Danish strain 1331)	0.9% NaCl saline	Single 0.1 mL intradermal injection	Hospitalization due to COVID-19​	PCR-confirmed COVID-19, RTIs, SARS-CoV-2 seropositivity, TB infection, injection site reactions	NM

Statistical Analysis

Meta-analysis was performed using Review Manager (RevMan) 5.4. Pooled mean differences (MD) were used to compare the duration of symptomatic illness (days with symptoms) between the BCG and placebo groups, while odds ratios (OR) were used to assess the incidence of symptomatic COVID-19, severe COVID-19, hospitalization, COVID-19 seropositivity, and serious adverse events. All pooled estimates were calculated with 95% confidence intervals (CIs) using a random-effects model due to the expected heterogeneity among studies. Heterogeneity was assessed using the I² statistic, with values above 50% indicating substantial heterogeneity. Sensitivity analyses were conducted by excluding studies one by one (leave one out) to evaluate the robustness of the results.

Results

Nine studies with a total number of 10,295 participants were included in the analysis (with 5,156 in the BCG group and 5,139 in the placebo group), to explore the protective role of the BCG vaccine against COVID-19 in HCWs.

Symptomatic COVID-19

**Figure 2:**
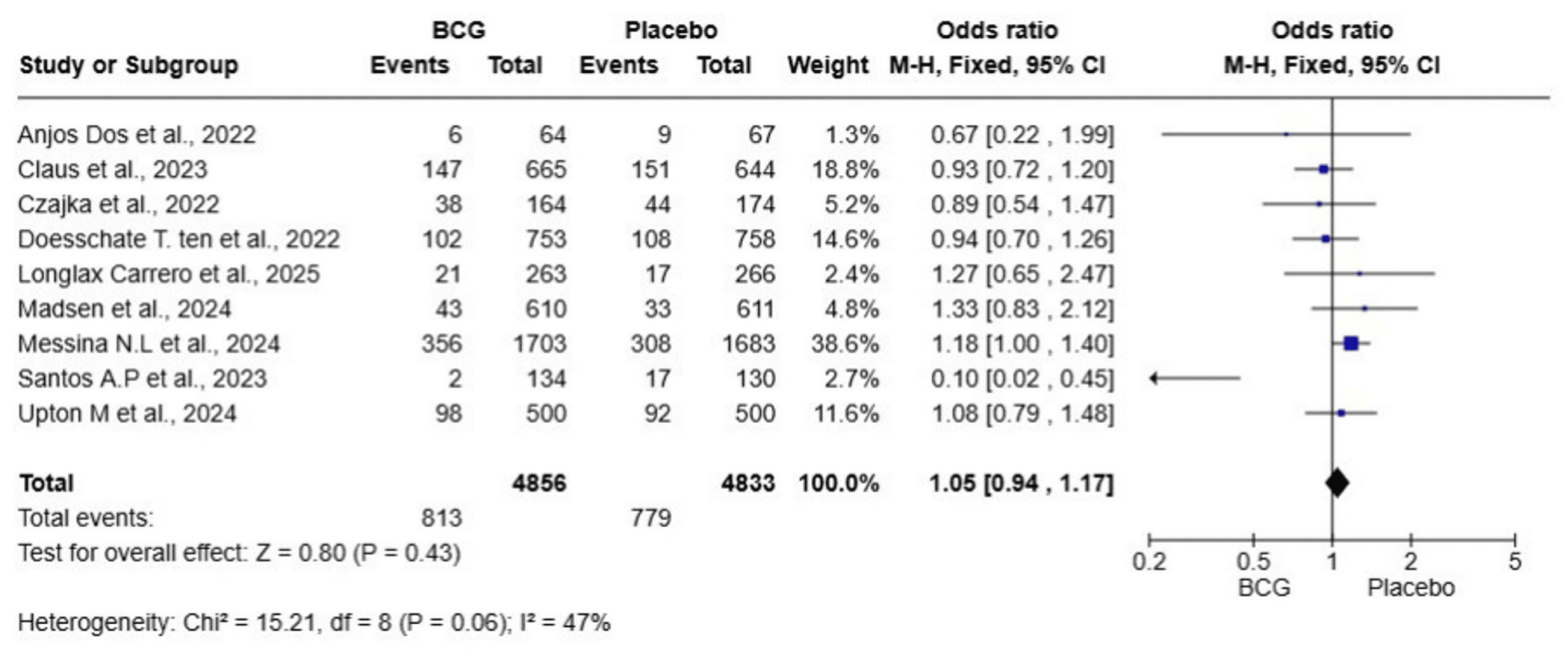
Forest plot of the effect of BCG vaccination versus placebo on the incidence of symptomatic COVID-19 among healthcare workers BCG: Bacillus Calmette-Guérin; COVID-19: coronavirus disease 2019; CI: confidence interval

A total of nine studies were included in this meta-analysis, encompassing 9,689 HCWs (4,856 in the BCG group and 4,833 in the placebo group). The pooled analysis revealed an OR of 1.05, 95% CI: 0.94-1.17, p = 0.43, indicating no statistically significant effect of BCG vaccination compared to placebo in preventing symptomatic COVID-19 among HCWs. Heterogeneity was moderate (I² = 47%), suggesting variability across the included studies (Figure 2). A sensitivity analysis excluding the study by Santos et al., 2023 [16], which was a notable outlier, resulted in an OR of 1.08 (95% CI: 0.96-1.21, p = 0.22), with heterogeneity substantially reduced (I² = 0%). This confirms the overall finding of no significant protective effect.

Severe COVID-19 Symptoms

**Figure 3:**
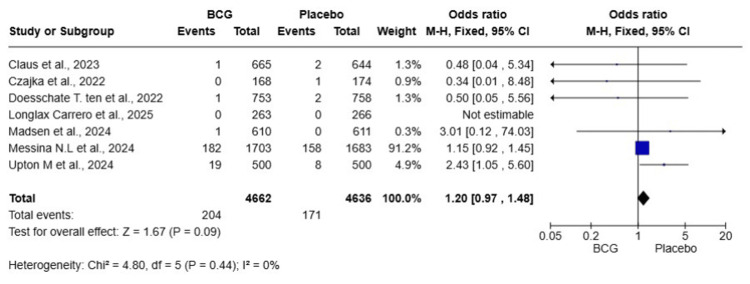
Forest plot of the effect of BCG vaccination versus placebo on the incidence of severe COVID-19 among healthcare workers BCG: Bacillus Calmette-Guérin; COVID-19: coronavirus disease 2019; CI: confidence interval

A total of seven studies were included in this meta-analysis, with 9,298 HCWs (4,662 in the BCG group and 4,636 in the placebo group). The pooled OR was 1.20 (95% CI: 0.97-1.48, p = 0.09), indicating that BCG vaccination did not significantly reduce the risk of severe COVID-19 compared to placebo. Heterogeneity was low (I² = 0%), suggesting that the included studies were relatively homogeneous (Figure 3).

Hospitalization

**Figure 4:**
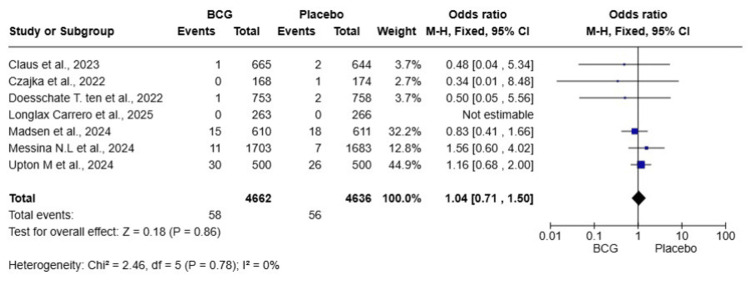
Forest plot of the effect of BCG vaccination versus placebo on COVID-19-related hospitalizations among healthcare workers BCG: Bacillus Calmette-Guérin; COVID-19: coronavirus disease 2019; CI: confidence interval

A total of seven studies were included in this meta-analysis, involving 9,298 HCWs (4,662 in the BCG group and 4,636 in the placebo group). The pooled OR was 1.04 (95% CI: 0.71-1.50, p = 0.86), indicating that BCG vaccination did not significantly reduce the risk of hospitalization due to COVID-19 compared to placebo. Heterogeneity was low (I² = 0%), suggesting that the results across studies were consistent (Figure 4).

Duration of Symptomatic COVID-19

**Figure 5:**
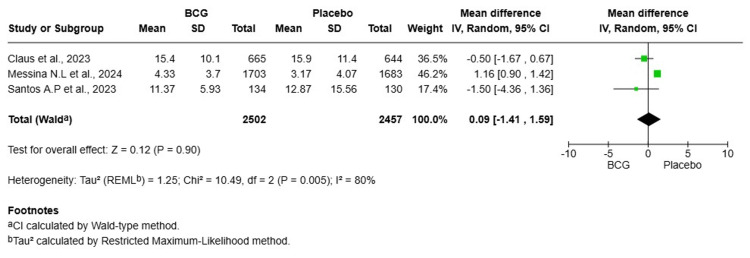
Forest plot of the effect of BCG vaccination versus placebo on the duration of symptomatic illness (days with symptoms) in healthcare workers BCG: Bacillus Calmette-Guérin; SD: standard deviation; CI: confidence interval

A total of three studies were included in this meta-analysis, involving 4,959 HCWs (2,502 in the BCG group and 2,457 in the placebo group). The pooled MD in the duration of symptomatic COVID-19 was 0.09 days (95% CI: -1.41-1.59, p = 0.90), indicating no significant difference in the number of symptomatic days between the BCG and placebo groups. Heterogeneity was high (I² = 80%), suggesting substantial variability among the included studies. To address this, a random-effects model was used (Figure 5). A sensitivity analysis excluding the study by Messina et al., 2024 [15], which appeared to be a key source of heterogeneity, resulted in a pooled mean difference of -0.70 days (95% CI: -1.88-0.48, p = 0.24), with heterogeneity completely resolved (I² = 0%). This confirms the primary finding of no statistically significant difference between the groups. 

COVID-19 Seropositivity

**Figure 6:**
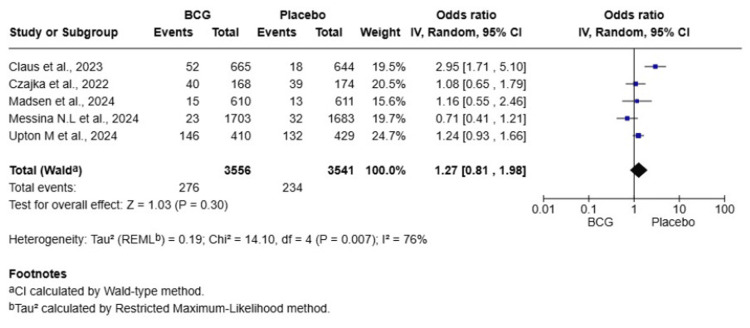
Forest plot of the effect of BCG vaccination versus placebo on COVID-19 seropositivity among healthcare workers BCG: Bacillus Calmette-Guérin; COVID-19: coronavirus disease 2019; CI: confidence interval

A total of five studies were included in this meta-analysis, involving 7,097 HCWs (3,556 in the BCG group and 3,541 in the placebo group). The pooled OR for COVID-19 seropositivity was 1.27 (95% CI: 0.81-1.98, p = 0.30), indicating no statistically significant difference in seropositivity rates between the BCG and placebo groups. Heterogeneity was high (I² = 76%), suggesting substantial variability among the included studies. A random-effects model was used to account for this heterogeneity (Figure 6). A sensitivity analysis excluding the study by Claus et al., 2023 [10], which was a major source of heterogeneity, resulted in an OR of 1.08 (95% CI: 0.92-1.27, p = 0.58), with heterogeneity substantially reduced (I² = 19%). This confirms that after removing the influential outlier, there is no evidence of an effect of BCG vaccination on seropositivity.

Serious Adverse Events

**Figure 7:**
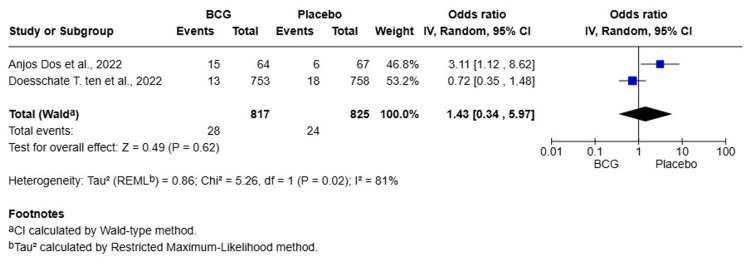
Forest plot of the effect of BCG vaccination versus placebo on the incidence of serious adverse events among healthcare workers BCG: Bacillus Calmette-Guérin; CI: confidence interval

A total of two studies were included in this meta-analysis, involving 1,642 HCWs (817 in the BCG group and 825 in the placebo group). The pooled OR for serious adverse events was 1.43 (95% CI: 0.34-5.97, p = 0.62), indicating no statistically significant difference in the occurrence of serious adverse events between the BCG and placebo groups. Heterogeneity was high (I² = 81%), reflecting substantial variability between the studies. A random-effects model was used to account for this (Figure 7).

Risk of Bias

**Figure 8:**
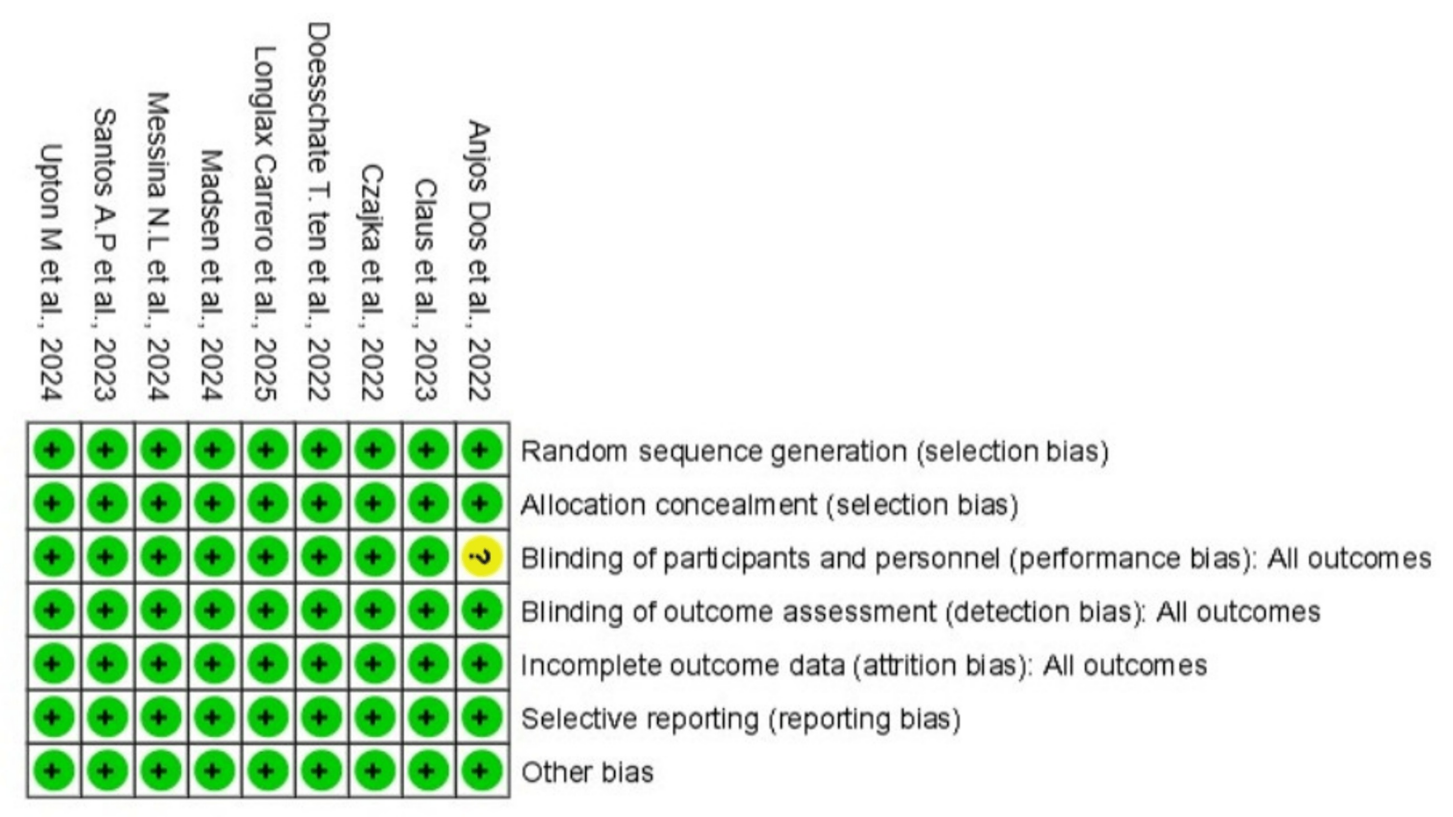
Risk of bias summary for the included studies evaluating BCG vaccination versus placebo in healthcare workers, using the Cochrane risk of bias tool BCG: Bacillus Calmette-Guérin

**Figure 9:**
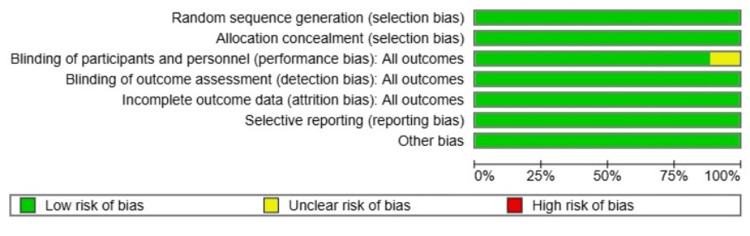
Risk of bias graph for the included studies evaluating BCG vaccination versus placebo in healthcare workers, using the Cochrane risk of bias tool BCG: Bacillus Calmette-Guérin

The risk of bias assessment showed that all studies had a low risk in most domains, including random sequence generation, blinding of participants and personnel, blinding of outcome assessment, incomplete outcome data, selective reporting, and other biases. However, Dos Anjos et al. [9] had unclear risk due to unclear data about the blinding for the healthcare participants; while researchers/statisticians were blinded, which may introduce some selection bias. Despite this, the overall methodological quality of the included studies was high, suggesting that the findings of the meta-analysis are robust and unlikely to be significantly affected by bias (Figures 8, 9).

Discussion

The COVID-19 pandemic has brought into focus the need for effective protective strategies among HCWs, who are at high risk of SARS-CoV-2 infection. The BCG vaccine, through its putative induction of nonspecific "trained immunity," was an early promising candidate for repurposing [19]. Small early observational studies fueled this hypothesis, suggesting an association between national BCG vaccination policies and improved COVID-19 outcomes [20]. However, the current meta-analysis of nine RCTs totaling more than 10,000 HCWs represents the highest level of evidence to date, indicating that BCG vaccination does not offer a protective effect against COVID-19 in this population.

This lack of efficacy is evident for the prevention of initial infection (symptomatic COVID-19), mitigation of disease severity (severe disease and hospitalization), and course of illness (duration of symptoms). The results for seropositivity, which were initially highly heterogeneous, converged to a null effect after sensitivity analysis, further reinforcing the absence of a biological signal. This comprehensive lack of effect strongly suggests that heterologous immunity induced by BCG is inadequate to alter the clinical course of SARS-CoV-2 infection in exposed adults.

While some reviews have suggested a potential benefit of BCG vaccination against COVID-19, as the one conducted by Gong et al. [21], our results provide no support for such an association in HCWs. Our findings are solidly corroborated by other recent and rigorous systematic reviews that, despite slight differences in their scope, show a consistent null effect. The meta-analysis by Wen et al. [22], which included nine RCTs, found that BCG vaccination did not reduce the infection rate of COVID-19 (OR = 0.96; 95% CI: 0.82-1.13), a result closely aligned with our finding for symptomatic COVID-19 (OR = 1.05, 95% CI: 0.94-1.17) [22]. Their nonsignificant results for COVID-19-related hospitalization (OR = 0.66, 95% CI: 0.37-1.18) and mortality (OR = 0.64; 95% CI: 0.17-2.44) reinforce our nonsignificant findings for hospitalization (OR = 1.04, 95% CI: 0.71-1.50) and severe disease (OR = 1.20, 95% CI: 0.97-1.48).

Most importantly, the review by Xia et al. [23] represents the most direct external validation of our work owing to their subgroup analysis on HCWs. They reported a RR of 1.03 (95% CI: 0.93-1.15) for COVID-19 in HCWs, remarkably close to the point estimate and certainty of our primary outcome [23]. By the same token, their null results for severe COVID-19 (RR = 1.25; 95% CI: 0.92-1.70) and hospitalization (RR = 0.93; 95% CI: 0.58-1.50) in the general population further reinforce our results [23]. Lastly, the review by Jain et al. [5], focusing on BCG revaccination specifically, also found no significant effect with respect to the incidence of COVID-19 infection (OR = 1.04; 95% CI: 0.91-1.19) or hospitalizations (OR = 0.81; 95% CI: 0.38-1.72) [5]. The convergence of these independent quantitative assessments, across different analytical approaches (ORs and RRs) and slightly different study inclusions (e.g., general population, revaccination), underscores the reliability of the current evidence that BCG does not confer a protective effect against COVID-19.

The heterogeneity observed in some of our analyses, such as for symptomatic duration and seropositivity. In general, sensitivity analyses showed this heterogeneity to be due to single outlying studies, such as Messina et al. [15] for symptom duration and Claus et al. [10] for seropositivity. When such outliers were excluded, the heterogeneity was completely resolved, and the pooled estimate invariably stabilized on a null effect. This suggests that the key conclusion of our meta-analysis is robust and not an artifact of conflicting data. High heterogeneity for serious adverse events, driven by two studies with opposite point estimates, prevents any conclusive safety finding and mandates the need for more data from large RCTs specifically powered for such outcomes, although the current data does not raise a red flag for major safety concerns.

The overall methodological quality of the included studies was high, and most domains had a low risk of bias. This strengthens the confidence in our pooled results, as they are derived from a robust body of evidence less likely to be affected by systematic errors. This meta-analysis of high-quality RCTs provides convincing evidence that BCG vaccination does not protect HCWs from SARS-CoV-2 infection or COVID-19 disease. The research priority should now shift away from further RCTs of BCG for COVID-19 prevention in adults and toward understanding the limits of trained immunity and identifying other nonspecific immune interventions. For public health decision-making, our results indicate that BCG vaccination should not be recommended or used for the purpose of preventing COVID-19.

Limitations

This meta-analysis has several limitations. First, the number of studies informing specific outcomes, such as the duration of symptomatic illness (n = 3), seropositivity (n = 5), and serious adverse events (n = 2), was limited; this reduces the precision of these pooled estimates, as reflected by wider CIs. Second, although the overall risk of bias was judged as low, one study had an unclear risk because of the non-blinding of healthcare professionals, which may introduce performance bias. Third, we observed moderate to high heterogeneity in analyses for symptomatic infection (I² = 47%), symptom duration (I² = 80%), seropositivity (I² = 76%), and serious adverse events (I² = 81%). Heterogeneity likely reflects true differences between study populations, local COVID-19 transmission dynamics, and BCG vaccine strains. However, extensive sensitivity analyses showed that heterogeneity was often due to a single outlier study, and exclusion of that study consistently strengthened the null effect without changing the overall conclusion. Finally, because this was a review restricted to HCWs, our findings may not be generalizable to other populations, such as older adults or immunocompromised populations.

## Conclusions

This meta-analysis of nine RCTs offers definitive proof that there is no protective effect of BCG vaccination against COVID-19 in HCWs. The most robust finding, from the largest pool of participants, was that BCG vaccination did not reduce symptomatic COVID-19 incidence (OR = 1.05, 95% CI: 0.94-1.17, p = 0.43). The following results were also not significant: severe COVID-19 (OR = 1.20, 95% CI: 0.97-1.48, P = 0.09); COVID-19-related hospitalization (OR = 1.04, 95% CI: 0.71-1.50, p = 0.86); SARS-CoV-2 seropositivity (OR = 1.27, 95% CI: 0.81-1.98, p = 0.30); and duration of symptomatic illness (MD = 0.09 days, 95% CI: -1.41-1.59, p = 0.90). Finally, rates of serious adverse events were also not significantly different from placebo (OR = 1.43, 95% CI: 0.34-5.97, p = 0.62). The consistent lack of effect across all endpoints, further supported by sensitivity analyses, underscores the robustness of these null findings. Accordingly, BCG vaccination should not be recommended or used as a strategy to prevent COVID-19 in HCWs.
